# Supplementary material for: Alzheimer’s Disease and circadian disruption sex-specifically contribute to a loss of bone maintenance in APP/PS1 model mice
Source: bioRxiv. 2026 May 5:2026.05.01.722089. Preprint. [Version 1] doi: 10.64898/2026.05.01.722089 (PMC13174557; doi:10.64898/2026.05.01.722089)
Supplement: Supplement 1 [file media-1.pdf]

Supplemental Table 1: 2-way ANOVA analysis and Fisher's Exact test on serum and brain Aβ42 ELISA results. Brain homogenate data was below the assay limit of detection (LOD) resulting in zero inflation, thus analyzed via contingency test (Fisher's Exact test) to determine significant genotype effect. The LOD contingency table is shown where Aβ42 detection is reported (Aβ42 – or Aβ42 +). Mean and standard deviation reported (top), test statistic (Fstat) p-value reported (bottom). Bolded red values highlight significant 2-way ANOVA result ($p<0.05$).

Aβ42 ELSIA		Condition	Serum	Brain		Aβ42 -	Aβ42 +
Mean±SD	Male	DS/LM	62.17 ±18.31	0 ±0		4	0
		DS/AP	219.7 ±67.44	47.81 ±14.18		0	4
		CD/LM	85.36 ±7.938	0 ±0		4	0
		CD/AP	196 ±60.41	102.6 ±19.56		0	4
2-way ANOVA	Lighting	Fstat	<0.001		Fisher's Exact (P value)	**2.00E-04**	
		p.val	0.987				
	Genotype	Fstat	75.680				
		p.val	**<0.0001**				
	Interaction	Fstat	2.315				
		p.val	0.138				
Mean±SD	Female	DS/LM	90.64 ±11.8	0 ±0		4	0
		DS/AP	200.1 ±59.83	86.4 ±50.29		0	4
		CD/LM	99.37 ±18.49	2.03 ±4.53		5	0
		CD/AP	213 ±43.89	145.8 ±213		0	4
2-way ANOVA	Lighting	Fstat	0.699		Fisher's Exact (P value)	**<0.0001**	
		p.val	0.409				
	Genotype	Fstat	74.820				
		p.val	**<0.0001**				
	Interaction	Fstat	0.025				
		p.val	0.875				

Supplemental Table 2: Repeated measures, mixed effects results testing for age and treatment effects in changing activity volume. Test statistic (Fstat) and p-value are reported. Bolded red values highlight significant mixed-effects result ($p<0.05$).

Mixed-effects analysis		Males	Females
**Age**	**Fstat**	20.610	4.788
	**p.val**	**0.0002**	**0.0407**
**Treatment**	**Fstat**	2.584	0.7470
	**p.val**	0.0791	0.5357
**Interaction**	**Fstat**	0.338	3.107
	**p.val**	0.798	**0.0496**

Supplemental Table 3: Mid-shaft cortical femur morphology assessed by micro-CT imaging. Results shown are mean and standard deviation for each group and parameter. Two-way ANOVA results shown, Fstat = calculated F statistic, p.val = p-value calculated. BA/TA% = bone area fraction, Ec.Pm = endocortical perimeter, Ps.Pm = periosteal perimeter, Ct.Th = cortical thickness. Bolded red values highlight significant 2-way ANOVA result (p<0.05).

Cortical Femur		Condition	Ct.Ar	Ct.Ar/Tt.Ar (%)	Ct.Th	Ec.Pm	Ps.Pm	Tt.Ar
Mean±SD	Male	DS/LM	1.293 ±0.186	61.315 ±3.162	0.28 ±0.029	3.557 ±0.211	5.645 ±0.326	2.104 ±0.238
		DS/AP	1.289 ±0.174	60.723 ±3.067	0.275 ±0.025	3.693 ±0.156	5.734 ±0.347	2.119 ±0.231
		CD/LM	1.309 ±0.211	60.342 ±4.727	0.277 ±0.036	3.653 ±0.223	5.774 ±0.345	2.163 ±0.235
		CD/AP	1.383 ±0.225	59.93 ±3.737	0.29 ±0.043	3.65 ±0.332	5.875 ±0.309	2.238 ±0.269
2-way ANOVA	Lighting	Fstat	0.693	0.486	0.312	0.112	1.473	1.197
		p.val	0.411	0.491	0.580	0.740	0.234	0.282
	Genotype	Fstat	0.275	0.157	0.109	0.680	0.725	0.314
		p.val	0.604	0.695	0.743	0.416	0.401	0.579
	Interaction	Fstat	0.344	0.005	0.702	0.743	0.003	0.136
		p.val	0.562	0.944	0.408	0.395	0.959	0.715
Mean±SD	Female	DS/LM	1.104 ±0.045	64.175 ±4.268	0.266 ±0.03	2.99 ±0.16	4.951 ±0.167	1.645 ±0.121
		DS/AP	1.049 ±0.083	63.242 ±1.934	0.262 ±0.011	3.042 ±0.21	4.944 ±0.238	1.66 ±0.153
		CD/LM	1.092 ±0.076	65.075 ±2.484	0.275 ±0.002	2.981 ±0.187	4.997 ±0.202	1.68 ±0.129
		CD/AP	1.117 ±0.1	63.585 ±2.3	0.263 ±0.002	3.019 ±0.28	5.101 ±0.098	1.756 ±0.067
2-way ANOVA	Lighting	Fstat	0.784	0.310	0.482	0.035	1.904	1.822
		p.val	0.385	0.583	0.496	0.853	0.182	0.191
	Genotype	Fstat	0.234	1.179	1.327	0.290	0.428	0.878
		p.val	0.633	0.289	0.264	0.595	0.520	0.359
	Interaction	Fstat	1.532	0.062	0.353	0.007	0.567	0.378
		p.val	0.229	0.805	0.559	0.934	0.459	0.545

Supplemental Table 4: Femoral head trabecular morphology assessed by µCT imaging. Results shown are mean and standard deviation for each group and parameter. Two-way ANOVA results shown, Fstat = calculated F statistic, p.val = p-value calculated. Conn.D. = Connectivity density, DA = Dynamic anisotropy, SMI = structural model index, Tb.N = Trabecular number, Tb.Pf = Trabecular pattern factor, Tb.Sp = Trabecular separation, Tb.Th = Trabecular thickness. Bolded red values highlight significant 2-way ANOVA result ($p<0.05$).

Femoral Head		Condition	BV/TV %	Conn.D (1/mm^3)	DA	SMI	Tb.N (1/mm)	Tb.Pf (1/mm)	Tb.Sp (mm)	Tb.Th (mm)
Mean±SD	Male	DS/LM	58.388 ±7.86	4430.154 ±831.891	3.47 ±1.153	0.699 ±0.264	6.735 ±0.89	9.011 ±4.553	0.095 ±0.023	0.087 ±0.011
		DS/AP	51.772 ±4.622	4151.766 ±490.045	2.469 ±0.492	1.057 ±0.194	6.257 ±0.5	11.325 ±1.944	0.091 ±0.015	0.083 ±0.002
		CD/LM	58.722 ±6.951	5241.896 ±1345.904	2.956 ±0.64	0.744 ±0.298	8.24 ±2.28	8.507 ±3.066	0.074 ±0.026	0.074 ±0.015
		CD/AP	51.853 ±8.464	4333.001 ±1200.782	2.127 ±0.238	1.297 ±0.454	6.74 ±1.225	14.731 ±6.233	0.081 ±0.024	0.077 ±0.007
2-way ANOVA	Lighting	Fstat	0.008	2.144	2.792	1.730	4.621	1.011	4.176	6.895
		p.val	0.931	0.153	0.105	0.198	**0.039**	0.323	**0.049**	**0.013**
	Genotype	Fstat	8.055	3.065	12.754	17.731	4.577	8.751	0.037	0.016
		p.val	**0.008**	0.090	**0.001**	**>0.001**	**0.040**	**0.006**	0.849	0.901
	Interaction	Fstat	0.003	0.864	0.112	0.811	1.220	1.835	0.464	0.885
		p.val	0.958	0.360	0.740	0.375	0.278	0.185	0.501	0.354
Mean±SD	Female	DS/LM	62.765 ±5.084	8845.434 ±1612.136	6.116 ±1.239	1.431 ±0.055	8.009 ±0.552	25.617 ±5.99	0.051 ±0.009	0.078 ±0.005
		DS/AP	66.249 ±9.255	11840.062 ±4710.012	4.895 ±0.485	1.966 ±0.251	8.255 ±1.354	32.568 ±3.52	0.037 ±0.011	0.081 ±0.003
		CD/LM	64.124 ±5.795	8765.438 ±1451.478	3.484 ±0.142	2.104 ±0.702	8.245 ±0.705	31.023 ±11.54	0.041 ±0.008	0.08 ±0.003
		CD/AP	69.247 ±3.726	9243.726 ±2079.384	5.529 ±1.298	1.654 ±0.352	9.033 ±0.498	25.596 ±7.001	0.036 ±0.004	0.077 ±0.005
2-way ANOVA	Lighting	Fstat	0.790	1.499	4.963	0.925	2.334	0.065	2.523	0.552
		p.val	0.383	0.233	**0.040**	0.348	0.140	0.801	0.127	0.465
	Genotype	Fstat	3.085	2.523	0.845	0.051	2.429	0.062	8.312	0.064
		p.val	0.092	0.126	0.371	0.824	0.133	0.806	**0.009**	0.802
	Interaction	Fstat	0.112	1.325	13.268	6.872	0.666	4.080	1.813	2.330
		p.val	0.741	0.262	**0.002**	**0.016**	0.423	0.056	0.192	0.141

Supplemental Table 5: Parameters from Raman spectroscopy and fluorescent advanced glycation end product (fAGE). Results shown are mean and standard deviation for each parameter. Two-way ANOVA results shown, Fstat = calculated F statistic, p-value = calculated p statistic. Crystallinity reported as inv. Phosphate band, Carbonate to phosphate ratio, mineral to matrix ratio (calculated with Amide I and Amide III peaks), carboxymethyl-lysine (CML), and fluorescent advanced glycation end product (fAGE) normalized to collagen content (mg).

Cortical Femur Molecular Parameters		Condition	Crystallinity 1/ν1PO43	Carbonate/ Phosphate (ν1CO32- /ν1PO43-)	Mineral/ Matrix ν2PO43- /Amide I	Mineral/ Matrix ν2PO43- /Amide III	Carboxylmethyl-lysine A1150/CH2-wag	fAGEs (ng of Quinine/mg of Collagen)
Mean±SD	Male	DS/LM	0.05 ±0.00038	0.21 ±0.02654	1.48 ±0.15926	13.18 ±2.801	0.07 ±0.01659	70.39 ±59.34114
		DS/AP	0.05 ±0.00059	0.23 ±0.01164	1.2 ±0.10058	10.63 ±2.68171	0.14 ±0.04505	466.38 ±337.75185
		CD/LM	0.05 ±0.00017	0.2 ±0.02212	1.04 ±0.28179	11.55 ±1.42442	0.12 ±0.03187	369.48 ±197.33318
		CD/AP	0.05 ±0.00076	0.23 ±0.06318	1.29 ±0.14583	10.49 ±2.20634	0.15 ±0.05064	363.27 ±159.68415
2-way ANOVA	Lighting	Fstat	0.123	0.005	0.532	4.028	2.205	0.844
		p.val	0.732	0.946	0.480	0.068	0.163	0.376
	Genotype	Fstat	0.011	1.301	2.208	0.048	7.029	3.338
		p.val	0.917	0.276	0.163	0.830	**0.021**	0.093
	Interaction	Fstat	0.183	0.110	0.371	9.042	0.779	3.555
		p.val	0.677	0.746	0.554	**0.011**	0.395	0.084
Mean±SD	Female	DS/LM	0.053 ±0.0004	0.221 ±0.0264	12.666 ±2.5792	1.23 ±0.2229	0.052 ±0.026	30.626 ±17.4791
		DS/AP	0.053 ±0.0004	0.233 ±0.0767	9.576 ±1.0483	0.968 ±0.344	0.12 ±0.0333	138.007 ±61.9243
		CD/LM	0.053 ±0.0008	0.21 ±0.0727	9.8 ±1.8036	0.963 ±0.1976	0.066 ±0.0385	91.668 ±84.6336
		CD/AP	0.053 ±0.0004	0.26 ±0.0507	9.93 ±2.2925	1.044 ±0.1575	0.149 ±0.0346	329.454 ±222.3762
2-way ANOVA	Lighting	Fstat	0.045	0.086	1.941	0.788	2.045	5.320
		p.val	0.834	0.773	0.183	0.388	0.172	**0.037**
	Genotype	Fstat	0.499	1.332	2.695	0.707	25.094	9.942
		p.val	0.490	0.265	0.120	0.413	**1.28E-04**	**0.007**
	Interaction	Fstat	0.015	0.515	3.186	2.523	0.238	1.419
		p.val	0.905	0.484	0.093	0.132	0.632	0.253

Supplemental Table 6: Notched three point bending strength test results. Data shown are mean and standard deviation for each group and parameter. Two-way ANOVA results shown, Fstat = calculated F statistic, p.val = p-value calculated. Bolded red values highlight significant 2-way ANOVA result ($p<0.05$).

Femur Strength Testing		Condition	Initiation Toughness Kc, in (MPa√m)	Maximum Toughness Kc, max (MPa√m)	Toughening Effect ΔK (MPa√m)
Mean±SD	Male	LM	0.74 ±0.09995	1.76 ±0.29265	1.02 ±0.29116
		AP	0.33 ±0.10815	0.94 ±0.44614	0.61 ±0.35975
		CD	0.23 ±0.04172	0.92 ±0.26741	0.69 ±0.24902
		CD/AP	0.18 ±0.05994	0.69 ±0.2113	0.51 ±0.18336
2-way ANOVA	Lighting	Fstat	69.964	13.100	2.686
		p.val	**1.37E-06**	**0.003**	0.125
	Genotype	Fstat	34.650	12.161	4.942
		p.val	**5.35E-05**	**0.004**	**0.045**
	Interaction	Fstat	21.341	3.826	0.733
		p.val	**4.80E-04**	0.072	0.407
Mean±SD	Female	LM	0.608 ±0.2093	1.811 ±0.4273	1.203 ±0.2951
		AP	0.368 ±0.0902	1.163 ±0.3588	0.795 ±0.2794
		CD	0.264 ±0.0477	0.723 ±0.1236	0.459 ±0.084
		CD/AP	0.231 ±0.0901	0.73 ±0.2906	0.499 ±0.2078
2-way ANOVA	Lighting	Fstat	19.815	27.899	24.621
		p.val	**4.66E-04**	**9.22E-05**	**1.71E-04**
	Genotype	Fstat	6.403	4.960	3.081
		p.val	**0.023**	**0.042**	0.100
	Interaction	Fstat	3.666	5.183	4.583
		p.val	0.075	**0.038**	**0.049**

Supplemental Table 7: Significant gene ontology (GO) terms for genes identified in the LRT analysis of aggregated bone marrow expression. Gene sets were submitted to GO analysis in clusters based on the hierarchical expression clustering as presented in Figure 3. Term: GO term name, Source: origin of GO term (BP: Biological process, CC: Cellular Component, MF: Molecular Function), Adjusted p-value: default method implemented by gprofiler2 package.

Cluster	Term	Source	Adjusted p-value	Gene Ratio
1	regulation of release of sequestered calcium ion into cytosol	GO:BP	2.56E-02	2/4
3	paraspeckles	GO:CC	1.71E-03	2/13
3	identical protein binding	GO:MF	1.37E-02	7/13
3	protein binding	GO:MF	4.42E-02	12/13
6	response to bacterium	GO:BP	1.62E-05	9/20
6	response to biotic stimulus	GO:BP	1.87E-05	11/20
6	calprotectin complex	GO:CC	8.53E-05	2/21
6	calcium-dependent protein binding	GO:MF	2.21E-04	4/21
6	response to other organism	GO:BP	2.32E-04	10/20
6	response to external biotic stimulus	GO:BP	2.37E-04	10/20
6	antimicrobial humoral response	GO:BP	2.93E-04	5/20
6	innate immune response	GO:BP	3.87E-04	8/20
6	biological process involved in interspecies interaction between organisms	GO:BP	5.15E-04	10/20
6	extracellular space	GO:CC	6.23E-04	9/21
6	response to external stimulus	GO:BP	7.57E-04	11/20
6	response to lipopolysaccharide	GO:BP	9.74E-04	6/20
6	response to molecule of bacterial origin	GO:BP	1.19E-03	6/20
6	cytoplasm	GO:CC	1.20E-03	19/21
6	defense response to symbiont	GO:BP	1.32E-03	8/20
6	ion binding	GO:MF	1.78E-03	15/21
6	regulation of transport	GO:BP	2.41E-03	9/20
6	antimicrobial humoral immune response mediated by antimicrobial peptide	GO:BP	2.79E-03	4/20
6	small molecule binding	GO:MF	2.90E-03	15/21
6	defense response to other organism	GO:BP	2.95E-03	8/20
6	humoral immune response	GO:BP	3.08E-03	5/20
6	defense response	GO:BP	3.74E-03	9/20
6	regulation of vesicle-mediated transport	GO:BP	4.39E-03	6/20
6	calcium ion binding	GO:MF	5.59E-03	6/21
6	response to stress	GO:BP	5.70E-03	12/20
6	neutrophil aggregation	GO:BP	7.46E-03	2/20
6	specific granule	GO:CC	7.72E-03	2/21
6	extracellular region	GO:CC	9.27E-03	9/21
6	regulation of inflammatory response	GO:BP	1.09E-02	5/20

6	cytolysis by host of symbiont cells	GO:BP	1.12E-02	2/20
6	defense response to bacterium	GO:BP	1.17E-02	5/20
6	programmed cell death	GO:BP	1.28E-02	9/20
6	cell death	GO:BP	1.28E-02	9/20
6	regulation of response to external stimulus	GO:BP	1.36E-02	7/20
6	regulation of localization	GO:BP	1.37E-02	9/20
6	antioxidant activity	GO:MF	1.45E-02	3/21
6	cytoplasmic vesicle	GO:CC	1.55E-02	8/21
6	intracellular vesicle	GO:CC	1.58E-02	8/21
6	response to lipid	GO:BP	1.79E-02	7/20
6	autocrine signaling	GO:BP	2.09E-02	2/20
6	vesicle	GO:CC	2.54E-02	8/21
6	positive regulation of cellular component organization	GO:BP	2.61E-02	7/20
6	positive regulation of intrinsic apoptotic signaling pathway	GO:BP	2.64E-02	3/20
6	regulation of defense response	GO:BP	3.05E-02	6/20
6	inflammatory response	GO:BP	3.47E-02	6/20
6	positive regulation of multicellular organismal process	GO:BP	3.69E-02	8/20
6	regulation of multicellular organismal process	GO:BP	3.99E-02	10/20
6	response to toxic substance	GO:BP	4.66E-02	4/20

Supplemental Table 8: Pairwise testing results of antioxidant genes comparing the experimental group to control group. Log2FC and Adjusted p-values are calculated using the FindMarkers (Seurat, v5) implementation of the MAST algorithm. Bolded red values indicate significant result (1.3<|Log2FC|) and adjusted p value <0.05). Grey boxes indicate comparisons not calculated due to low transcript abundance or pre-filtering of Log2FC(|Log2FC| < 0.1).

Antioxidant Genes	Gene Sub-group annotation	APvControl		CDvControl		CD/APvControl	
		Log2FC	Adjusted p value	Log2FC	Adjusted p value	Log2FC	Adjusted p value
Als2	ROS Stress Response	0.12	1.00	0.16	1.00	-0.30	1.00
Apc	Peroxidase			-0.12	1.00	-0.27	1.00
Apoe	ROS Stress Response	-1.23	1.00	-0.49	1.00	**-2.23**	**8.34E-17**
Ctsb	Peroxidase	-0.20	1.00			-0.20	1.00
Cyba	NADPH Oxidase					0.16	0.86
Ehd2	Peroxiredoxin	-0.17	1.00			-0.85	1.00
Gpx1	Glutathione	-0.38	2.22458E -24	0.12	5.95E-11		
Gpx4	Glutathione			-0.12	1.00	0.17	1.00
Gsr	Glutathione	**2.98**	**3.68E-286**	0.21	1.00	**1.97**	**8.71E-133**
Kif9	Peroxidase	0.63	1.00	-0.24	1.00	-0.12	1.00
Mpo	Peroxidase	**-1.51**	**1.87E-08**	-0.29	1.00	-0.81	1.00
Ncf2	NADPH Oxidase	0.15	1.00	0.21	3.06E-11	-0.21	1.00
Nox1	NADPH Oxidase	0.43	1.00	0.14	1.00	0.19	1.00
Nxn	Thioredoxin	0.22	1.00	0.26	1.00	-0.17	1.00
Prdx1	Peroxiredoxin	-0.42	2.21E-03	-0.23	1.00	0.19	1.00
Prdx2	Peroxiredoxin	-0.46	1.00			0.39	0.80
Prdx3	Peroxiredoxin	-0.27	1.00				
Prdx6	Peroxiredoxin			0.17	1.00	-0.23	1.00
Ptgs2	Peroxiredoxin	-0.27	1.00			-0.72	1.00
Rag2	Peroxiredoxin	0.19	1.00	-0.21	1.00	0.72	1.00
Scd1	Metabolic Redox Enzyme	-0.85	0.01	-0.13	1.00	-0.64	0.13
Serpinb1a	Antioxidant			0.42	8.02E-05	0.12	1.00
Slc41a3	Peroxidase	-0.22	1.00	0.22	1.00	0.11	1.00
Sod1	Superoxide Dismutase					-0.15	1.00
Txnip	ROS Stress Response					0.17	1.00

Supplemental Table 9: Significant differentially expressed genes (DEGs, as compared to control group) for each condition and the overlap between DEGs across the conditions. Enriched Gene Ontology (GO) for biological processes shown for unique gene expression sets. Intersection Set: transcripts contributing to GO terms. Adjusted p-value: default method implemented by clusterProfiler package.

Intersection Group	Transcripts	GO ID	Description	Intersection Set	Adjusted p-value	Gene Ratio
Shared across all	*Hspa1b, Hspa1a, Egr1*					
Unique to AP	*Gsr, Colec12, Dhx40, Nme7, Wrn, AA465934, Gm12166, Inpp4b, Slc15a2, Cdk5rap1, Chil3, Zfp985, Gm18870, Gm4070, Hpgd, Lgals3bp, Gm42031*	GO:0005044	scavenger receptor activity	*Lgals3bp/Colec12*	0.0036	2/13
		GO:0008408	3'-5' exonuclease activity	*Nme7/Wrn*	0.0096	2/13
		GO:0038024	cargo receptor activity	*Lgals3bp/Colec12*	0.0107	2/13
		GO:0004527	exonuclease activity	*Nme7/Wrn*	0.0107	2/13
		GO:0004386	helicase activity	*Dhx40/Wrn*	0.0243	2/13
Unique to CD/AP	*Pde4d, Gm11361, Dusp1, Apoe, Fabp5, Gm26532, Rgcc, Ppfia4, Apoc2, Ppp1r15a, Sirpb1c, Hmga1b, Adssl1, Glo1, Rnase2a, Gm50022, Gm10503, Ccdc180, Pmaip1, Btla, Ccl2, Adgrg5, Gm36560, Ckb, Slc20a1, Rwdd2a, Coq10b, Tppp3, H2-DMb2, Smim8, Ets2, Mafb, Trim36, Trib1*	GO:0001933	negative regulation of protein phosphorylation	*Pde4d/Apoe/Dusp1/Ppp1r15a/Trib1*	0.0102	5/29
		GO:0042326	negative regulation of phosphorylation	*Pde4d/Apoe/Dusp1/Ppp1r15a/Trib1*	0.0102	5/29
		GO:0071830	triglyceride-rich lipoprotein particle clearance	*Apoe/Apoc2*	0.0102	2/29
		GO:0034384	high-density lipoprotein particle clearance	*Apoe/Apoc2*	0.0102	2/29
		GO:0010563	negative regulation of phosphorus metabolic process	*Pde4d/Apoe/Dusp1/Ppp1r15a/Trib1*	0.0102	5/29
Unique to CD	*Hbegf, S100a8, S100a9, Vcan, Ngp, Mmp8, B930036N10Rik, Lcn2, Wfdc21, Hist1h2bc, Hist1h2ac, Gm13814, Hist1h2br, Hist1h3d, Gm11290, Hist2h4, Hist1h2bg, Vill, Gm26887, D830025C05Rik, Hist1h2bn, Camp, Hist2h2be, Hist1h4n, Hist1h2ah, Gm37168, Gm49692, Frmd8os*	GO:0018119	peptidyl-cysteine S-nitrosylation	*S100a8/S100a9*	0.0022	2/14
		GO:0017014	protein nitrosylation	*S100a8/S100a9*	0.0022	2/14
		GO:0032496	response to lipopolysaccharide	*S100a8/S100a9/Mmp8/Wfdc21*	0.0022	4/14
		GO:0070486	leukocyte aggregation	*S100a8/S100a9*	0.0022	2/14
		GO:0002237	response to molecule of bacterial origin	*S100a8/S100a9/Mmp8/Wfdc21*	0.0022	4/14
AP & CD	*Jun, Rhob, Gm49980, Gm43305, Tex14*					
CD/AP & AP	*Gm10260, Emp1, Hebp1, Gm42047, Nrxn1*					
CD/AP & CD	*Klf2, Hist1h4m, Tmem108*					

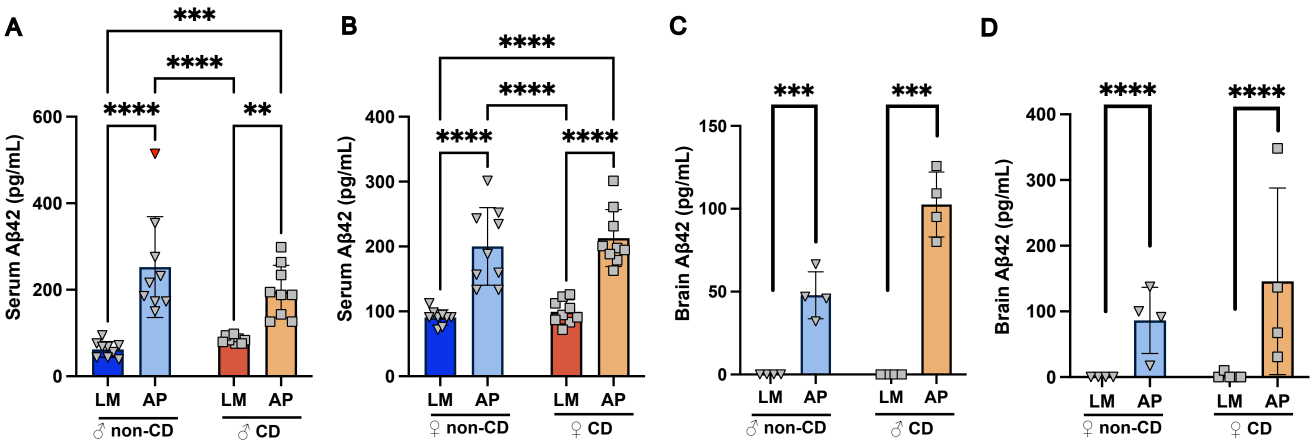

Supplemental Figure 1: APP/PS1 mouse (AP) show significant increase of amyloid beta 42 in the circulation and brain tissue. Amyloid beta 42 quantified via ELISA in **A)** male serum, **B)** female serum, Tukey's post-hoc pairwise comparison displayed (* p<0.05, **p<0.01, ***p<0.001, ****p<0.0001). **C)** male brain homogenate, **D)** female brain homogenate. In LM brain homogenate, values were below assay limit of detection, significance indicates results from Fisher's exact test based on frequency (detected vs. not detected) (* p<0.05, **p<0.01, ***p<0.001, ****p<0.0001). Dark blue = control/littermates (LM), Light blue = Alzheimer's genotype (AP), Dark orange = Circadianly disrupted (CD), and Light orange = CD/AP.

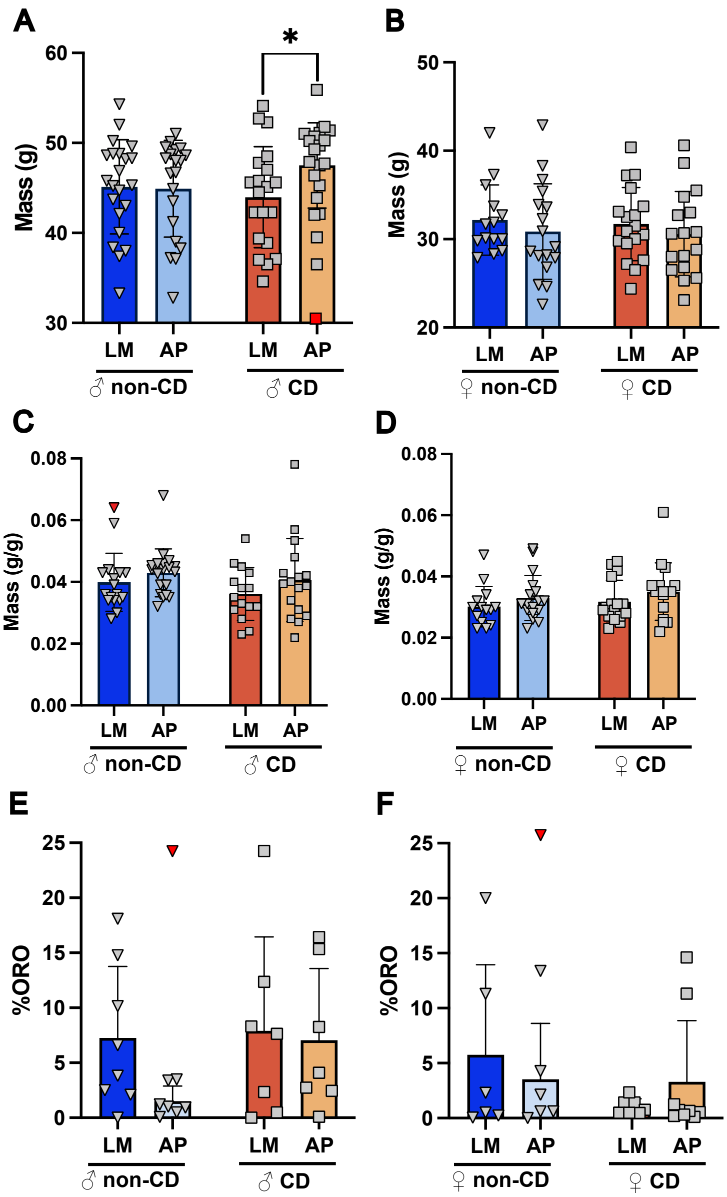

Supplemental Figure 2: Body mass, liver mass, and liver lipids show no signs of metabolic effects due to CD and AP. **A)** Male and **B)** Female body mass at the end of the 22-week protocol. **C)** Male and **D)** female liver mass normalized to body mass at the end of the 22-week protocol. **E)** Male and **F)** female quantification of liver lipids by cryosectioning and staining with Oil Red O. Tukey's post-hoc pairwise comparison displayed (* $p<0.05$), Red points indicate outlier removed from analysis as determined by Grubb's test. Dark blue = control/littermates (LM), Light blue = Alzheimer's genotype (AP), Dark orange = Circadianly disrupted (CD), and Light orange = CD/AP.

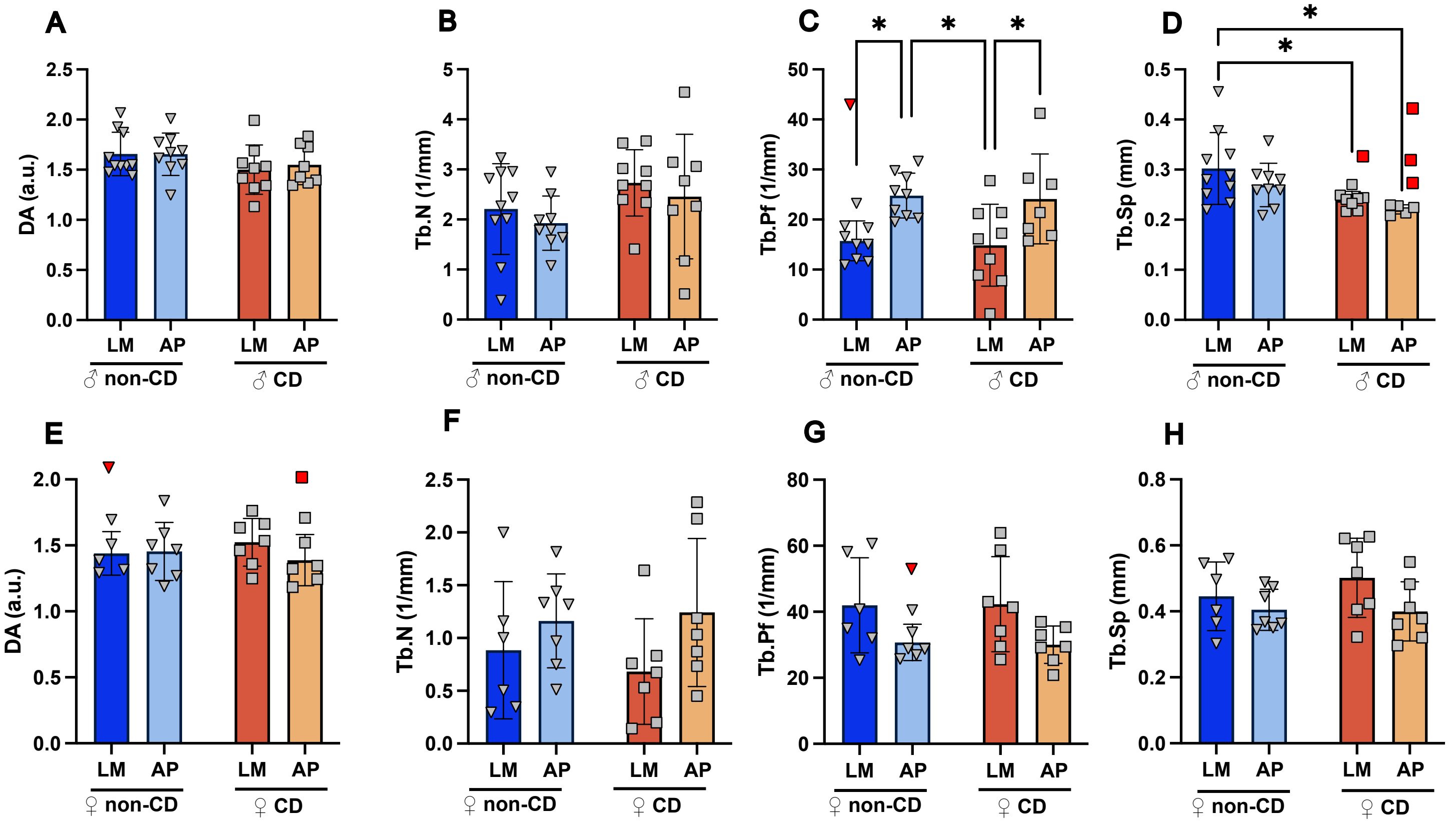

Supplemental Figure 3: Distal femur µCT parameters are sex-specifically affected by AP and CD. Male A) DA = Dynamic Anisotropy, B) Tb.N = Trabecular Number, C) Tb.Pf = Trabecular Pattern Factor, and D) Tb.Sp = Trabecular Separation by genotype and lighting condition. Female E) DA, F) Tb.N, G) Tb.Pf, and H) Tb.Sp by genotype and lighting condition. Tukey's post-hoc pairwise comparison displayed (* $p<0.05$). Red points indicate outlier removed from analysis as determined by Grubb's test. Dark blue = control/littermates (LM), Light blue = Alzheimer's genotype (AP), Dark orange = Circadianly disrupted (CD), and Light orange = CD/AP.

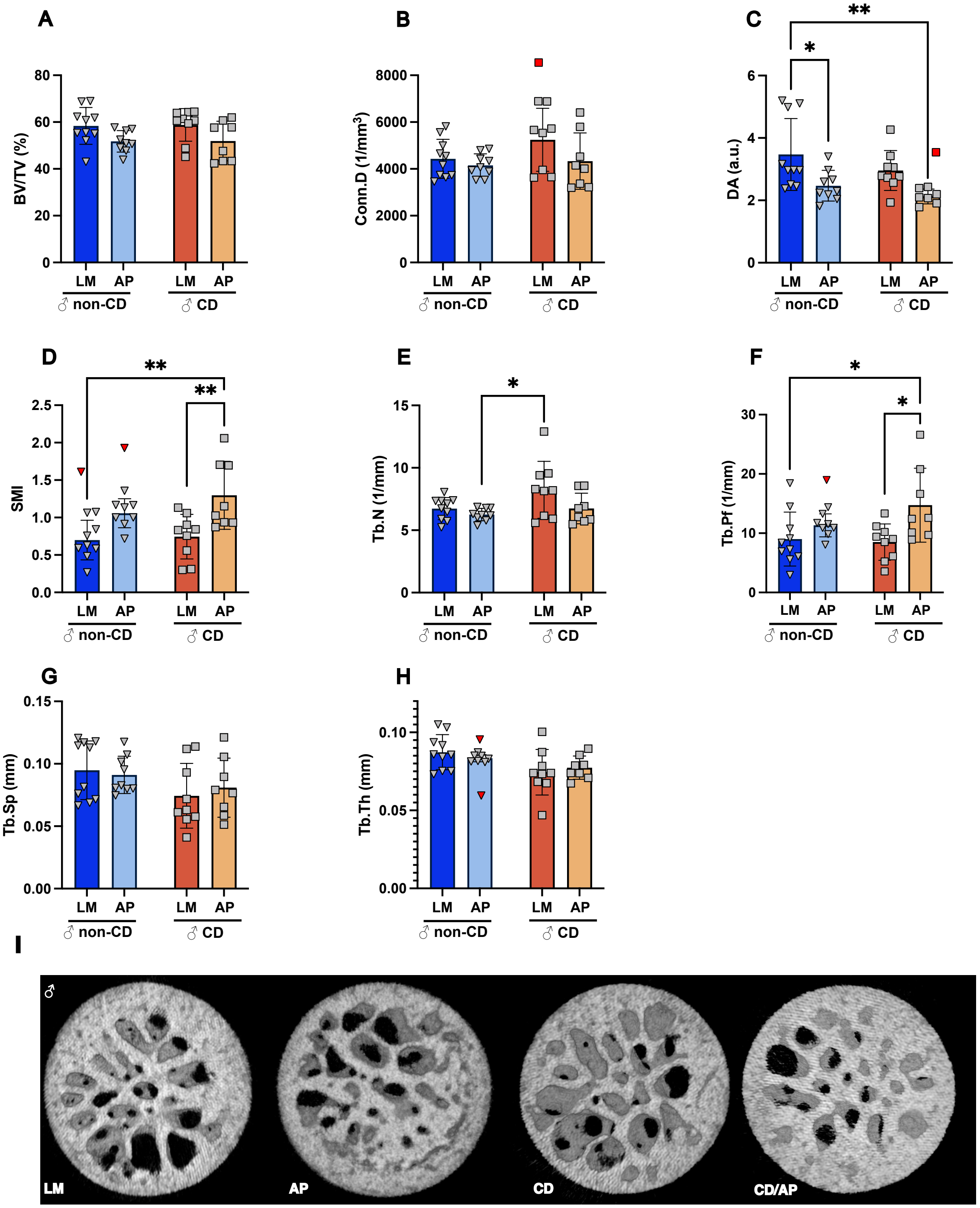

Supplemental Figure 4: Male femoral head trabecular morphology is affected by AP. The A) Bone volume fraction (BV/TV%), B) Connectivity density (Conn.D), C) Degree of Anisotropy (DA), D) Structural model index (SMI), E) Trabecular Number (Tb.N), F Trabecular Pattern Factor (Tb.Pf), G) Trabecular Separation (Tb.Sp), and H) Trabecular Thickness (Tb.Th) of the male femoral head trabeculae. I) Representative reconstruction of volume of interest in femoral head from male animals. Tukey's post-hoc pairwise comparison displayed (* $p<0.05$, ** $p<0.01$). Red points indicate outlier removed from analysis as determined by Grubb's test. Dark blue = control/littermates (LM), Light blue = Alzheimer's genotype (AP), Dark orange = Circadianly disrupted (CD), and Light orange = CD/AP.

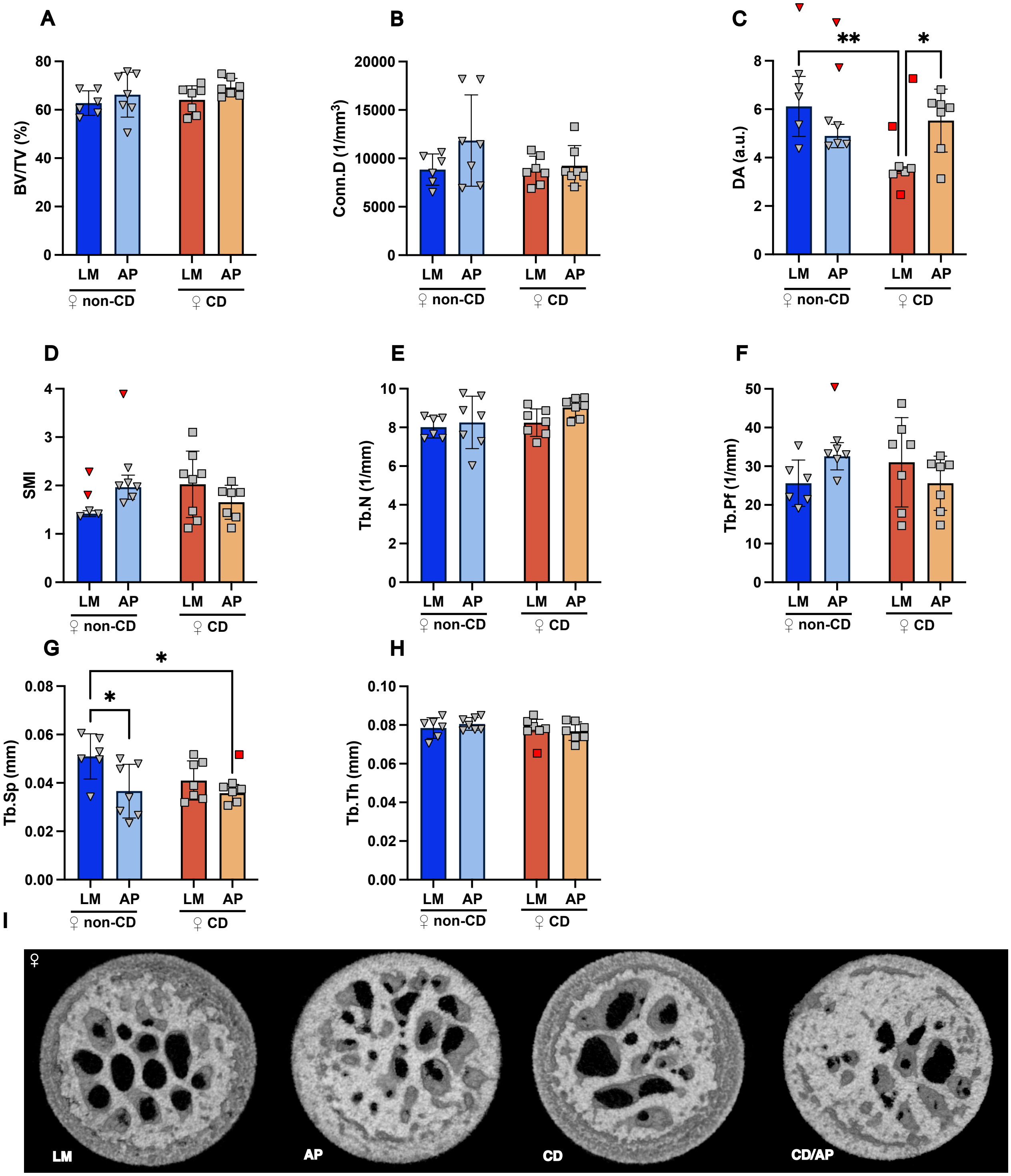

Supplemental Figure 5: Female femoral head trabecular morphology is affected by AP. The A) Bone volume fraction (BV/TV%), B) Connective density (Conn.D), C) Degree of Anisotropy (DA), D) Structural model index (SMI), E) Trabecular Number (Tb.N), F Trabecular Pattern Factor (Tb.Pf), G) Trabecular Separation (Tb.Sp), and H) Trabecular Thickness (Tb.Th) of the female femoral head trabeculae. I) Representative reconstruction of volume of interest in femoral head from female animals. Tukey's post-hoc pairwise comparison displayed (* $p<0.05$, ** $p<0.01$). Red points indicate outlier removed from analysis as determined by Grubb's test. Dark blue = control/littermates (LM), Light blue = Alzheimer's genotype (AP), Dark orange = Circadianly disrupted (CD), and Light orange = CD/AP.

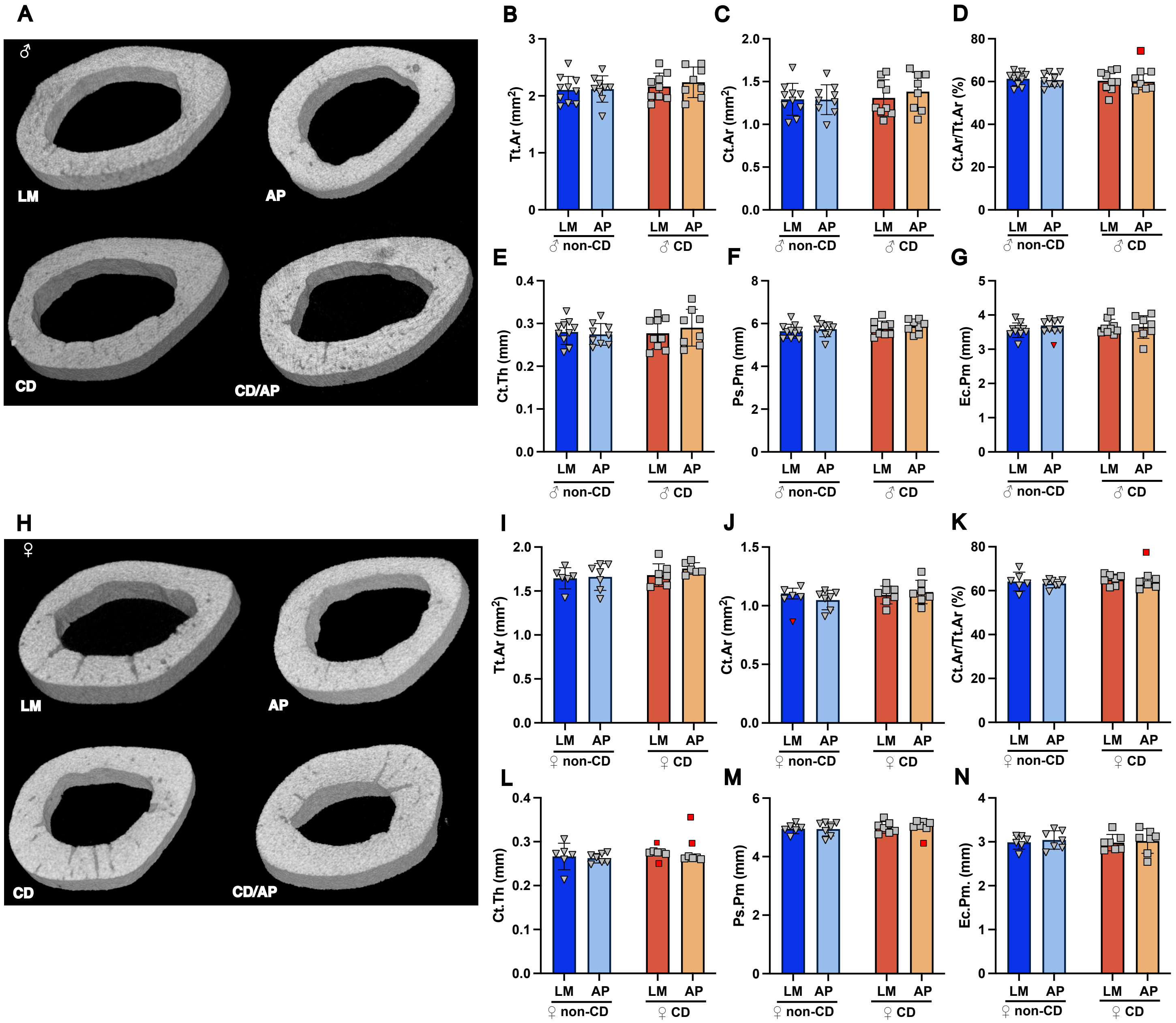

Supplemental Figure 6: Mid-shaft cortical bone shows no effect of AP and CD. Cortical bone morphology in male mice: A) Example reconstruction for male cortical volume of interest, B) Total cross sectional area inside the periosteal envelope (Tt.Ar), C) Cortical bone area (Ct.Ar), D) Cortical area fraction (Ct.Ar/Tt.Ar) E) Cortical thickness (Ct.Th) F) Periosteal Perimeter (Ps.Pm). G) Endocortical perimeter (Ec.Pm). Cortical bone morphology in female mice: H) Example reconstruction for female cortical volume of interest, I) Total cross sectional area inside the periosteal envelope (Tt.Ar), J) Cortical bone area (Ct.Ar), K) Cortical area fraction (Ct.Ar/Tt.Ar) L) Cortical thickness (Ct.Th) M) Periosteal Perimeter (Ps.Pm). N) Endocortical perimeter (Ec.Pm). Tukey's post-hoc pairwise comparison displayed (* $p<0.05$). Red points indicate outlier removed from analysis as determined by Grubb's test. Dark blue = control/littermates (LM), Light blue = Alzheimer's genotype (AP), Dark orange = Circadianly disrupted (CD), and Light orange = CD/AP.

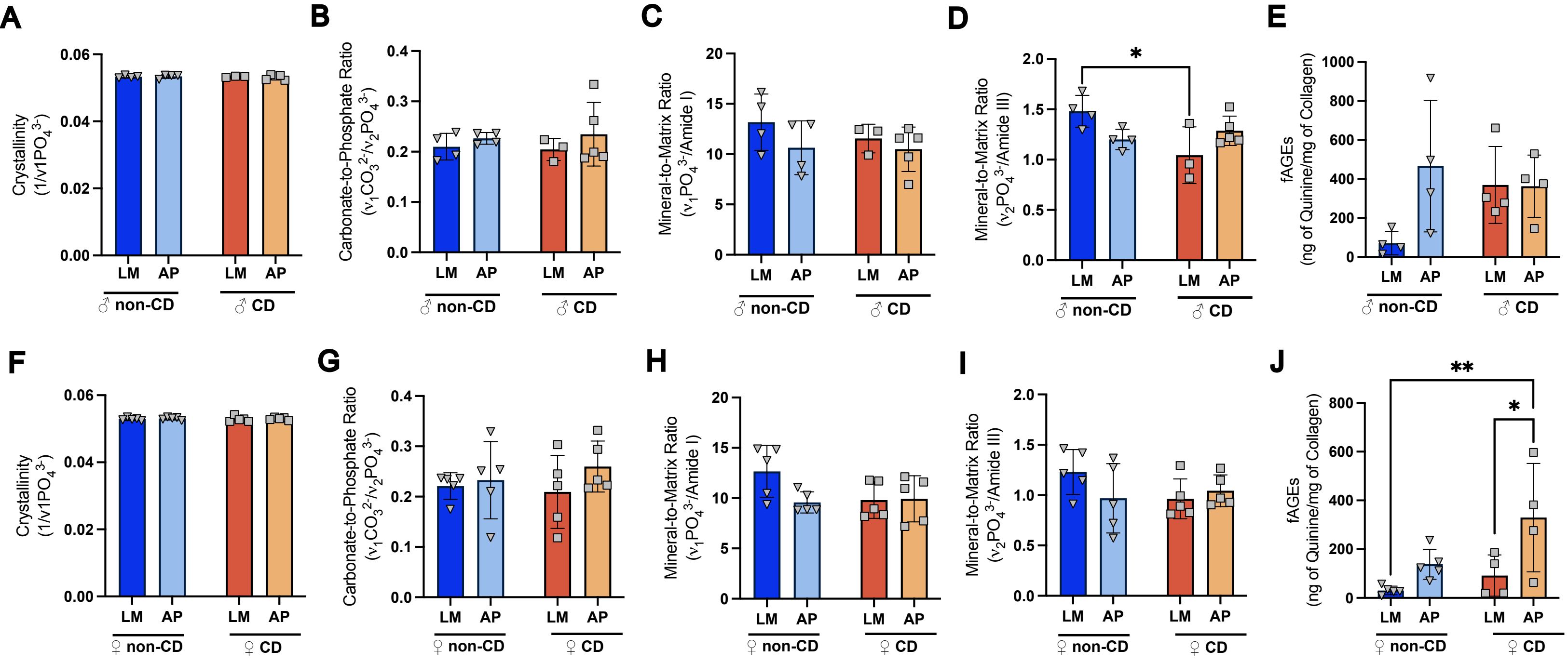

Supplemental Figure 7: Genotype and lighting condition have a sex-specific effect on fAGEs in cortical bone. The results of Raman spectroscopy and fluorescent AGE quantification. A) crystallinity, B) carbonate to phosphate ratio, C) mineral to matrix ratio-Amide 1, D) mineral to matrix ratio-Amide 3, and E) fAGE levels for male bones. F) crystallinity, G) carbonate to phosphate ratio, H) mineral to matrix ratio-Amide 1, I) mineral to matrix ratio-Amide 3, and J) fAGE levels for female bones. Dark blue = control/littermates (LM), Light blue = Alzheimer's genotype (AP), Dark orange = Circadianly disrupted (CD), and Light orange = CD/AP.

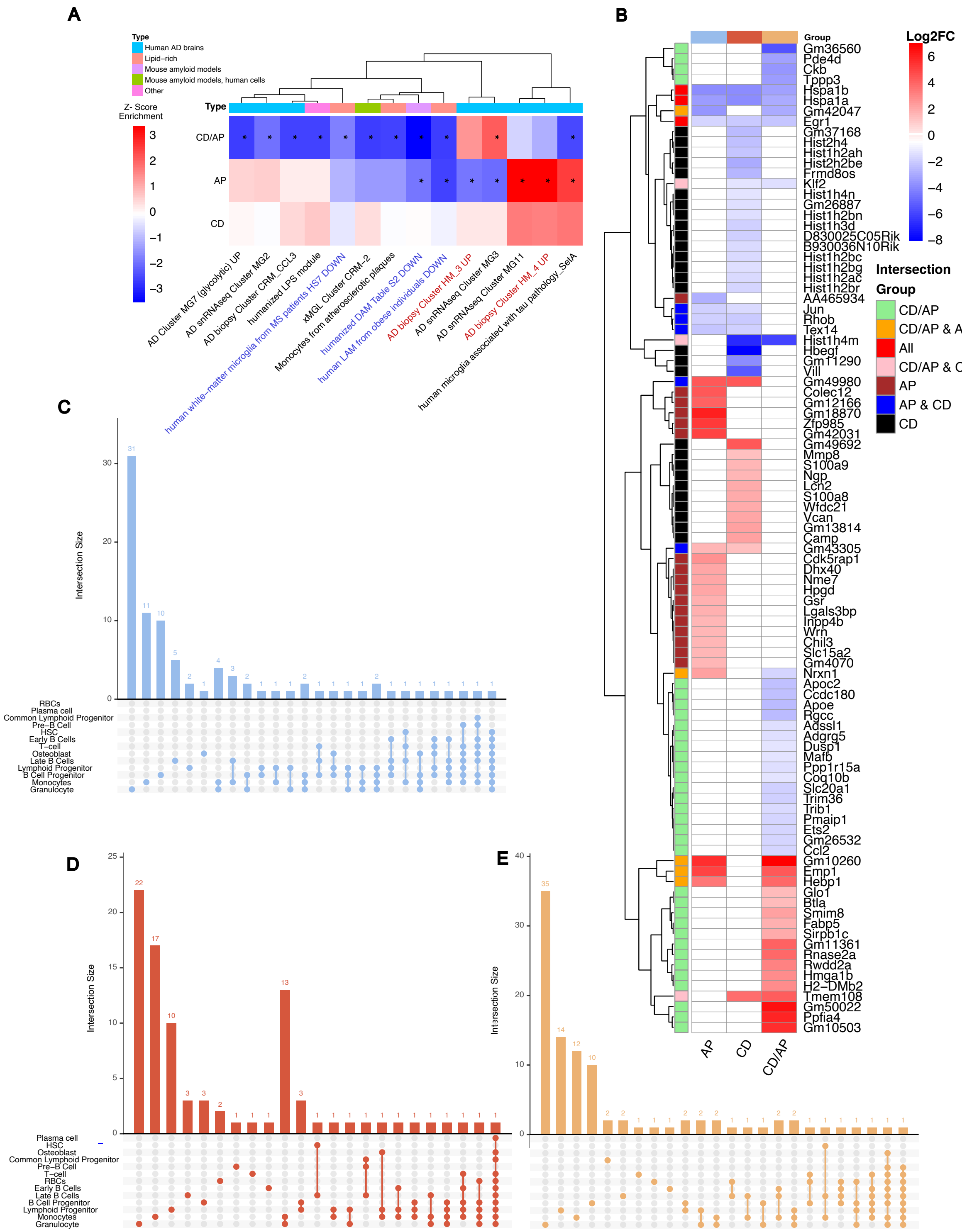

Supplemental Figure 8: DEGs are cell type specific to AP and CD; AP monocytes are enriched for Alzheimer's transcriptomic programs. A) GSEA enrichment using the MAST implementation, comparing monocyte enrichment to Alzheimer's patient transcriptomic gene sets derived from Goate et. al, **B)** Monocyte DEG Log2FC, white indicates no significant change. **C)** The overlap DEGs between cell types visualized with each cell type plotted with UpsetR (v1.4.0) Light blue = Alzheimer's genotype (AP) VS LM, **D)** Dark orange = Circadianly disrupted (CD) vs LM, and **E)** Light orange = CD/AP vs LM.

Supplemental Datasets

Marker genes used to identify cell clusters, cross referenced with CellxGene database.

Cluster	Gene	Name
B Cell Progenitor	Bach2	BTB and CNC homology, basic leucine zipper transcription factor 2
	Vpreb1	V-set pre-B cell surrogate light chain 1
	Lef1	lymphoid enhancer binding factor 1
	Cecr2	CECR2, histone acetyl-lysine reader
	Vpreb3	V-set pre-B cell surrogate light chain 3
CLP	Igll1	immunoglobulin lambda-like polypeptide 1
	Lef1	lymphoid enhancer binding factor 1
	Dntt	deoxynucleotidyltransferase, terminal
	Gfra2	glial cell line derived neurotrophic factor family receptor alpha 2
Early B Cells	Ms4a1	Membrane Spanning 4-Domains A1
	Cd79a	CD79A antigen (immunoglobulin-associated alpha)
	Bank1	B cell scaffold protein with ankyrin repeats 1
	Ly6d	lymphocyte antigen 6 family member D
Granulocyte	Lta4h	leukotriene A4 hydrolase
	Chil3	chitinase-like 3
	Camp	Cathelicidin Antimicrobial Peptide
	Ltf	lactotransferrin
HSC	Cd34	CD34 antigen
	Gata2	GATA binding protein 2
Late B Cells	H2-Eb1	histocompatibility 2, class II antigen E beta
	H2-Aa	histocompatibility 2, class II antigen A, alpha
Lymphoid Progenitor	Top2a	topoisomerase (DNA) II alpha
	Bcl7a	B cell CLL/lymphoma 7A
	Il7r	interleukin 7 receptor
	Pclaf	PCNA clamp associated factor
	Ezh2	enhancer of zeste 2 polycomb repressive complex 2 subunit
HSC	Cd34	CD34 antigen
	Gata2	GATA binding protein 2
Macrophage/ Dendritic Cells	Itgam	integrin alpha M
	Anpep	alanyl aminopeptidase, membrane
	Cd33	CD33 molecule
	Itgax	integrin alpha X
	Retnlg	resistin like gamma
	Mmp9	matrix metallopeptidase 9

	Ngp	neutrophilic granule protein
	Ltf	lactotransferrin
Monocytes	F13a1	coagulation factor XIII, A1 subunit
	Lgals1	lectin, galactose binding, soluble 1
	Ccr2	C-C motif chemokine receptor 2
Osteoblast	Kmo	kynurenine 3-monooxygenase
	Bst2	bone marrow stromal cell antigen 2
	Siglech	sialic acid binding Ig-like lectin H
	Runx2	runt related transcription factor 2
Plasma	Jchain	immunoglobulin joining chain
	Rplp1	ribosomal protein lateral stalk subunit P1
	Rplp41	Ribosomal Protein L41
Pre-B Cell	Ebf1	early B cell factor 1
	Bach2	BTB and CNC homology, basic leucine zipper transcription factor 2
	Aff3	AF4/FMR2 family, member 3
	Pax5	paired box 5
	Foxp1	forkhead box P1
RBCs	Hbb-bt	hemoglobin, beta adult t chain
	Hbb-bs	hemoglobin, beta adult s chain
	Hemgn	hemogen
	Sox6	SRY (sex determining region Y)-box 6
T Cells	Cd3e	CD3 antigen, epsilon polypeptide
	Cd4	CD4 antigen
	Cd8a	CD8 subunit alpha
	Il7r	interleukin 7 receptor
